# Updates on malaria incidence and profile in Malaysia from 2013 to 2017

**DOI:** 10.1186/s12936-020-3135-x

**Published:** 2020-01-31

**Authors:** Narwani Hussin, Yvonne Ai-Lian Lim, Pik Pin Goh, Timothy William, Jenarun Jelip, Rose Nani Mudin

**Affiliations:** 1 Clinical Research Centre, Hospital Taiping, Ministry of Health, 34000 Taiping, Perak Malaysia; 2 Department of Parasitology, Faculty of Medicine, University of Malaya, Kuala Lumpur, Malaysia; 3 Institute of Clinical Research, National Institute of Health, Ministry of Health, Setia Alam, Selangor Malaysia; 4 Gleneagles Hospital, Kota Kinabalu, Sabah Malaysia; 5 Malaria Control Unit, Disease Control Division, Ministry of Health, Putrajaya, Malaysia; 6 Vector Borne Disease Control Sector, Disease Control Division, Ministry of Health, Putrajaya, Malaysia

**Keywords:** Malaria, Peninsular Malaysia, Sabah and Sarawak, Incidence, Mortality, Case fatality rate

## Abstract

**Background:**

To date, most of the recent publications on malaria in Malaysia were conducted in Sabah, East Malaysia focusing on the emergence of *Plasmodium knowlesi*. This analysis aims to describe the incidence, mortality and case fatality rate of malaria caused by all *Plasmodium* species between Peninsular Malaysia and East Malaysia (Sabah and Sarawak) over a 5-year period (2013–2017).

**Methods:**

This is a secondary data review of all diagnosed and reported malaria confirmed cases notified to the Ministry of Health, Malaysia between January 2013 and December 2017.

**Results:**

From 2013 to 2017, a total of 16,500 malaria cases were notified in Malaysia. The cases were mainly contributed from Sabah (7150; 43.3%) and Sarawak (5684; 34.4%). Majority of the patients were male (13,552; 82.1%). The most common age group in Peninsular Malaysia was 20 to 29 years (1286; 35.1%), while Sabah and Sarawak reported highest number of malaria cases in age group of 30 to 39 years (2776; 21.6%). The top two races with malaria in Sabah and Sarawak were Bumiputera Sabah (5613; 43.7%) and Bumiputera Sarawak (4512; 35.1%), whereas other ethnic group (1232; 33.6%) and Malays (1025; 28.0%) were the two most common races in Peninsular Malaysia. *Plasmodium knowlesi* was the commonest species in Sabah and Sarawak (9902; 77.1%), while there were more *Plasmodium vivax* cases (1548; 42.2%) in Peninsular Malaysia. The overall average incidence rate, mortality rate and case fatality rates for malaria from 2013 to 2017 in Malaysia were 0.106/1000, 0.030/100,000 and 0.27%, respectively. Sarawak reported the highest average incidence rate of 0.420/1000 population followed by Sabah (0.383/1000). Other states in Peninsular Malaysia reported below the national average incidence rate with less than 0.100/1000.

**Conclusions:**

There were different trends and characteristics of notified malaria cases in Peninsular Malaysia and Sabah and Sarawak. They provide useful information to modify current prevention and control measures so that they are customised to the peculiarities of disease patterns in the two regions in order to successfully achieve the pre-elimination of human-only species in the near future.

## Background

Malaria poses a major public health challenge globally despite intensive effort to abate the disease. In 2017, the World Health Organization (WHO) estimated 219 million malaria cases and 435,000 malaria deaths worldwide [1]. Since the establishment of the Malaria Eradication Programme in 1967 in Malaysia, numbers of malaria cases have decreased significantly. Drastic reduction in the number of malaria cases from 243,870 in 1961 to 44,226 cases was reported in 1980 [2]. Subsequently, the programme was further reinforced and improved, resulting in 2302 malaria cases reported in 2016 [3]. Currently, Malaysia has been identified by WHO as one of the 21 countries having the potential to eliminate the human-only species by 2020.

Even though the number of malaria cases has reduced markedly, there is still a need to improve the malaria control and elimination programme as malaria is still a public health issue, especially in the hinterland and among aborigine groups who stay in the less developed parts of Malaysia. Furthermore, the influx of foreign workers from malaria-endemic countries and the challenge of resistance of malaria parasites to anti-malarial drugs heighten the threat of re-emergence of the disease.

Malaysia consists of Peninsular Malaysia and East Malaysia which comprises of Sabah and Sarawak. To date, most of the recent publications on malaria in Malaysia were conducted in Sabah, East Malaysia and these studies focused on the emergence of *Plasmodium knowlesi*, the fifth human *Plasmodium* species [4–8]. This present analysis aims to look into the incidence, mortality and case fatality rate of malaria caused by all *Plasmodium* species in the whole of Malaysia, which includes Peninsular Malaysia and East Malaysia, over a 5 year period (2013–2017). Specifically, this report describes the sociodemographic profiles of malaria cases in Peninsular Malaysia and East Malaysia from 2013 to 2017 and compares the different malaria trends between Peninsular Malaysia and East Malaysia (Sabah and Sarawak). In view of the pre-elimination status, which will be in 2020, this analysis is vital to identify differences in current malaria trends as well as to provide useful information to modify current prevention and control measures so that they are tailored to the peculiarities of disease patterns in the two regions of Malaysia.

## Methods

### Data collection

This is a secondary data review of all diagnosed and reported malaria confirmed cases (as defined [9] in Table 1) notified to the Ministry of Health, Malaysia between January 2013 and December 2017. The Malaysian Prevention and Control of Infectious Diseases Act 1988 states that it is compulsory for all laboratory-confirmed cases of malaria to be notified to the nearest District Health Office within 7 days of confirmed diagnosis. The State Health Department compiles all data for the whole state before sending the data to central national level.

**Table 1:** Case definitions for malaria [9]

Malaria case, confirmed	Malaria case (or infection) in which the parasite has been detected in a diagnostic test, i.e., microscopy, a rapid test or a molecular diagnostic test
Malaria, all cases	All malaria cases irrespective of *Plasmodium* species (including imported case)
Malaria case, imported	Malaria case or infection in which the infection was acquired outside the area in which it is diagnosed (get infected outside Malaysia)
Malaria case, indigenous	A case contracted locally with no evidence of importation and no direct link to transmission from an imported case (get infected in Malaysia)
Malaria elimination	Interruption of local transmission (reduction to zero incidence of indigenous case) of a specified malaria parasite in a defined geographical area as a result of deliberate activities. Continued measures to prevent re-establishment of transmission are required

Data extracted included population demography data, such as gender, age, ethnicity/race, and nationality. Other data included year of notification, state, type of infection, type of parasites, and outcome (alive or death). All data were treated as categorical variables. Age was collected as numerical variable in years but was later categorized into age groups of 10-year intervals. Ethnicity was based on the major ethic groups available in Malaysia. For states, the category included all 13 states and 2 federal territories in Malaysia. These were subsequently categorized into two groups based on locality, which included: (a) Peninsular Malaysia—consisted of 11 states and 1 federal territory (Federal Territory of Kuala Lumpur); and, (b) East Malaysia—consisted of Sabah, Sarawak and 1 federal territory (Federal Territory of Labuan). Types of infection were classified as defined [9] in Table 2.

**Table 2:** Case classification for malaria [9]

Import A case	Malaria case or infection in which the infection was acquired outside the area in which it is diagnosed. ‘A’ refers to infection from a country outside Malaysia
Indigenous case	A case contracted locally with no evidence of importation and no direct link to transmission from an imported case
Induced	A case the origin of which can be traced to a blood transfusion or other form of parenteral inoculation of the parasite but not to transmission by a natural mosquito-borne inoculation
Introduced	A case contracted locally, with strong epidemiological evidence linking it directly to a known imported case (first-generation local transmission)
Relapse	Malaria case attributed to activation of hypnozoites of *Plasmodium vivax* or *Plasmodium ovale* acquired previously

### Data analysis

The analysis was done using Microsoft Excel 2010. Summary descriptive statistics using frequency and percentage of characteristics documented for malaria cases were tabulated to obtain a clear understanding of the population studied. Bar and line graphs were used to show the trend of malaria over the years. Incidence and mortality rates were calculated based on the number of population in Malaysia for the respective year based on census from Department of Statistics [10]. Incidence rate was reported per 1000 population (IR)/1000 while mortality rate was reported per 100,000 population (MR)/100,000. Case fatality rate was reported as percentage (CFR) (%).

## Results

### Number of malaria cases

#### Overall

In the 5-year period 2013 to 2017, a total of 16,500 malaria cases were notified in Malaysia (Table 3). The number of notified malaria cases rose from 3850 in 2013 to 3923 in 2014 and then decreased to 2311 and 2302 cases in 2015 and 2016, respectively. The highest number of cases were notified in 2017 (n = 4114).

**Table 3:** Sociodemographic characteristics of malarial cases by Peninsular Malaysia and Sabah and Sarawak from 2013 to 2017

Variable	Peninsular Malaysia		Sabah and Sarawak		Total	
	No	%	No	%	No	%
Year						
2013	1240	32.2	2610	67.8	3850	100.0
2014	843	21.5	3080	78.5	3923	100.0
2015	394	17.1	1917	82.9	2311	100.0
2016	521	22.6	1781	77.4	2302	100.0
2017	667	16.2	3447	83.8	4114	100.0
Total	3665	22.2	12,835	77.8	16,500	100.0
Gender						
Female	605	16.5	2341	18.2	2946	17.8
Male	3058	83.4	10,494	81.8	13,552	82.1
NA	2	0.05	–	–	2	0.01
Age group (year)						
0–9	190	5.2	377	2.9	567	3.4
10–19	468	12.8	1470	11.5	1938	11.7
20–29	1286	35.1	2612	20.4	3898	23.6
30–39	904	24.7	2776	21.6	3680	22.3
40–49	439	12.0	2621	20.4	3060	18.5
50–59	245	6.7	1908	14.9	2153	13.0
60–69	87	2.4	722	5.6	809	4.9
70–79	41	1.1	278	2.2	319	1.9
80–89	4	0.1	57	0.4	61	0.4
90+	–	–	14	0.1	14	0.1
NA	1	0.03	–	–	1	0.01
Race						
Malay	1025	28.0	225	1.8	1250	7.6
Chinese	133	3.6	594	4.6	727	4.4
Indian	56	1.5	6	0.05	62	0.4
BP Sabah	25	0.7	5613	43.7	5638	34.2
BP Sarawak	25	0.7	4512	35.1	4537	27.5
Indigenous^a^	549	15.0	1	0.01	550	3.3
Others	1232	33.6	1199	9.3	2431	14.7
Foreigner	620	16.9	685	5.3	1305	7.9
*Plasmodium* species						
*P. knowlesi*	1478	40.3	9902	77.1	11,380	69.0
*P. vivax*	1548	42.2	1177	9.2	2725	16.5
*P. falciparum*	603	16.4	1011	7.9	1614	9.8
*P. malariae*	7	0.2	542	4.2	549	3.3
*P. ovale*	9	0.3	62	0.5	71	0.4
*Mixed*	20	0.5	141	1.1	161	1.0
Case classification						
Import A	1698	46.3	1218	9.5	2916	17.7
Indigenous	1905	52.0	11,582	90.2	13,487	81.7
Induced	6	0.2	3	0.02	9	0.05
Introduced	50	1.4	3	0.02	53	0.3
Relapse	6	0.2	29	0.2	35	0.2
Outcome status						
Alive	3641	99.4	12,813	99.8	16,454	99.7
Death	24	0.6	22	0.2	46	0.3

*NA* not available, *BP* Bumiputera/local Pribumis

^a^i.e., Orang Asli

#### Sabah and Sarawak

Table 3 shows that each year, Sabah and Sarawak reported higher number of malaria cases than Peninsular Malaysia. The trend was very much like that in overall Malaysia. The number of notified malaria cases increased from 2610 (67.8%) in 2013 to 3080 (78.5%) in 2014 before it declined to 1917 (82.9%) in 2015 and 1781 (77.4%) in 2016. The highest reported number of malaria cases in Malaysia in 2017 was mainly contributed from the increase in notified malaria cases in Sabah and Sarawak which was 3447 (83.8%).

During the 5 years (2013 to 2017), the total number of malaria cases were mainly contributed from notified malaria cases in Sabah (7150; 43.3%), followed by Sarawak (5684; 34.4%) as shown in Table 4. Further details in Table 4 shows that consecutively from 2013 to 2017, Sabah reported the highest number of malaria cases, followed by Sarawak except for 2016. Every year (excluding 2016), the malaria cases in Sabah contributed to more than 40% of total malaria cases in Malaysia. However in 2016, Sarawak outnumbered Sabah with the highest number of notified malaria cases in the whole Malaysia with 1064 (46.2%) cases. Sabah reported the lowest percentage of malaria cases in 2013 (41.7%; 1606) and the highest in 2017 (48.7%; 2004). Whilst in Sarawak, the percentage of malaria cases was highest in 2015 (37.8%, 873) and lowest in 2013 (26.1%; 1004).

**Table 4:** Number of malaria cases and deaths according to year and state in Malaysia from 2013 to 2017

State	Year										Total	
	2013		2014		2015		2016		2017			
	No	%	No	%	No	%	No	%	No	%	No	%
Sabah	1606 (3)	41.7	1779 (4)	45.4	1044 (1)	45.2	717	31.2	2004 (4)	48.7	7150 (12)	43.3
Sarawak	1004 (3)	26.1	1301 (1)	33.2	873 (2)	37.8	1064 (1)	46.2	1442 (3)	35.1	5684 (10)	34.4
Selangor	301 (1)	7.8	326 (2)	8.3	100	4.3	91	4.0	105 (2)	2.6	923 (5)	5.6
Kelantan	377 (3)	9.8	129	3.3	54	2.3	111	4.8	168	4.1	839 (3)	5.1
Perak	101	2.6	81	2.1	34 (2)	1.5	184	8.0	130 (2)	3.2	530 (4)	3.2
Pahang	179 (1)	4.6	128 (1)	3.3	79 (1)	3.4	40	1.7	85 (1)	2.1	511 (4)	3.1
Johor	61 (1)	1.6	42	1.1	45	2.0	41	1.8	81	2.0	270 (1)	1.6
Negeri Sembilan	54 (1)	1.4	32 (1)	0.8	18	0.8	11 (1)	0.5	36	0.9	151 (3)	0.9
Penang	39	1.0	37	0.9	17	0.7	3	0.1	9	0.2	105	0.6
Kedah	46	1.2	19	0.5	14	0.6	9	0.4	16 (1)	0.4	104 (1)	0.6
Terengganu	41	1.1	17	0.4	11 (1)	0.5	9	0.4	22	0.5	100 (1)	0.6
FT of Kuala Lumpur	32	0.8	20	0.5	16 (1)	0.7	15	0.6	12	0.3	95 (1)	0.6
Melaka	8	0.2	10	0.2	2	0.1	6	0.3	2	0.05	28	0.2
Perlis	1 (1)	0.03	2	0.05	4	0.2	1	0.04	1	0.02	9 (1)	0.1
FT of Labuan	–	–	–	–	–	–	–	–	1	0.02	1	0.01
Total	3850 (14)	100	3923 (9)	100	2311 (8)	100	2302 (2)	100	4114 (13)	100	16,500 (46)	100

*FT* federal territory

() number of death

Unlike Sabah and Sarawak, the Federal Territory of Labuan did not report any cases of malaria from 2013 to 2016. It only notified 1 case (0.01%) in 2017.

#### Peninsular Malaysia

In Peninsular Malaysia, a different trend was observed. Table 3 shows that the number of notified malaria cases reduced from 1240 (32.2%) in 2013 to 843 (21.5%) in 2014 and declined further to 394 (17.1%) cases in 2015. However, it increased to 521 (22.6%) in year 2016 and 667 (16.2%) in 2017.

Table 4 shows that the other states in Peninsular Malaysia reported much lower numbers of malaria cases compared to Sabah and Sarawak. For total number of malaria cases within 5 years, five states reported a range of 1.6% (270 cases in Johor), 3.1% (511 cases in Pahang), 3.2% (530 cases in Perak), 5.1% (839 cases in Kelantan) to 5.6% (923 cases in Selangor) of the total number of malaria cases in Malaysia. The other six states and one federal territory reported less than 1.0% of the total notified malaria cases in Malaysia. They were Negeri Sembilan (0.9%; 151), Penang (0.64%; 105), Kedah (0.63%; 104), Terengganu (0.61%; 100), Federal Territory of Kuala Lumpur (0.58%; 95), Melaka (0.2%; 28) and Perlis (0.1%; 9).

Among all the states and federal territory in Peninsular Malaysia, Kelantan recorded the highest number of notified malaria cases in 2013 (377; 9.8%) and 2017 (168; 4.1%). In 2014 and 2015, the highest number of malaria cases in Peninsular Malaysia was reported in Selangor (326; 8.3% in 2014 and 100; 4.3% in 2015) whereas Perak notified the highest number of malaria cases in 2016 (184; 8.0%). The least number of notified malaria cases was in Perlis for 2013 (1: 0.03%), 2014 (2; 0.05%), 2016 (1; 0.04%) and 2017 (1; 0.02%). Melaka reported the smallest number of malaria case in 2015 with 2 cases (0.1%).

### Malaria cases according to sociodemographic characteristics (gender, age, and race)

#### Overall

According to gender, 13,552 (82.1%) cases were found in male patients (Table 3). The commonest age group was 20–29 years (3898; 23.6%) followed by 30–39 years (3680; 22.3%), 40–49 years (3060; 18.5%), 50–59 years (2153; 13.0%) and 10–19 years (1938; 11.7%). Each of the other age groups reported less than 5.0% of the total cases. Overall in Malaysia, malaria was most common among Bumiputera Sabah (5638; 34.2%), followed by Bumiputera Sarawak (4537; 27.5%). Malay contributed only 1250 (7.6%) of the total malaria cases, lesser than other ethnic groups (2431; 14.7%) and foreigners (1305; 7.9%).

#### Sabah and Sarawak

Similar proportion of male malaria patients were reported in Sabah and Sarawak (10,494; 81.8%). Sabah and Sarawak reported highest number of malaria cases in older age group of 30 to 39 years (2776; 21.6%). This was followed by the age group of 40–49 years with 2621 cases (20.4%), 20–29 years (2612; 20.4%), 50–59 years (1908; 14.9%) and 10–19 years (1470; 11.5%). Malaria was diagnosed in 5613 (43.7%) of Bumiputera Sabah and 4512 (35.1%) of Bumiputera Sarawak. They were followed by others (1199; 9.3%), foreigners (685, 5.3%), Chinese (594; 4.6%) and Malay (225; 1.8%).

#### Peninsular Malaysia

There were 3058 (83.4%) of notified malaria cases in Peninsular Malaysia who were male. The most common age group was 20 to 29 years (1286; 35.1%). Subsequently, the next common age group was 30–39 years (904; 24.7%), 10–19 years (468; 12.8%) and 40–49 years (439; 12.0%). Other ethnic group and Malays were the two most common races in Peninsular Malaysia which contributed 33.6% (1232) and 28.0% (1025), respectively. The other common groups were foreigners (620; 16.9%) and indigenous people (549; 15.0). Each Bumiputera Sabah and Bumiputera Sarawak only accounted for 25 (0.7%) of the cases.

### Malaria cases causative agent according to *Plasmodium* species

#### Overall

Table 3 shows that *P. knowlesi* is the most common causative agent for malaria, comprising 69.0% (11,380) of the malaria cases in Malaysia, followed by *P. vivax* (16.5%, 2725), *Plasmodium falciparum* (9.8%, 1614), *Plasmodium malariae* (3.3%, 549), *Plasmodium ovale* (0.4%, 71) and mixed infections (1.0%, 161).

#### Sabah and Sarawak

The highest number of *P. knowlesi* in Malaysia was contributed mainly from *P. knowlesi* cases notified in Sabah and Sarawak which was 9902 (77.1%). Other types of *Plasmodium* species only accounted for less than 10% of the total malaria cases. There were 1177 (9.2%) *P. vivax*, 1011 (7.9%) *P. falciparum*, 542 (4.2%) *P. malariae*, 62 (0.5%) *P. ovale*, and 141 (1.1%) mixed infection.

**Fig. 1:**
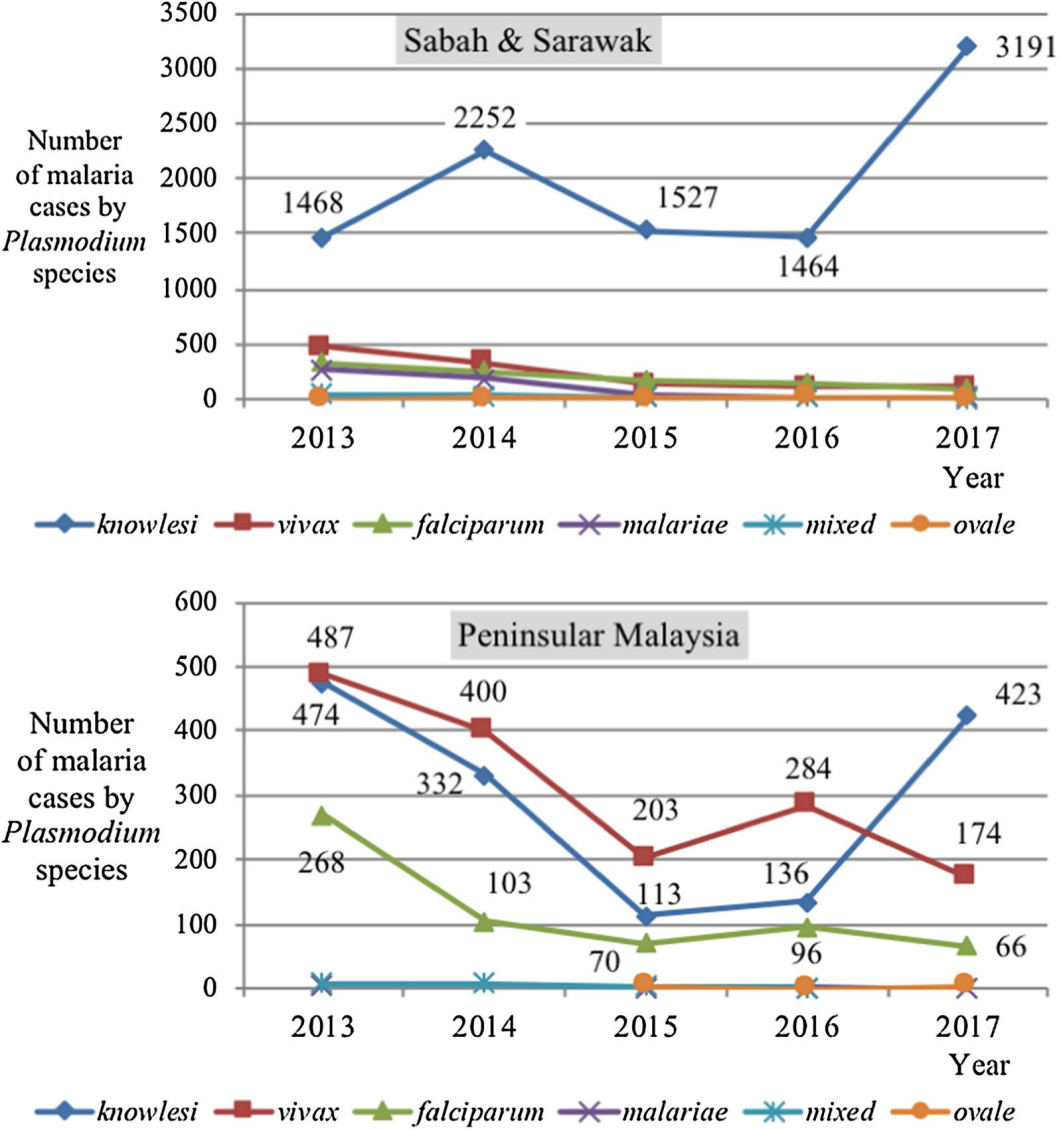
Number of malaria cases according to year and *Plasmodium* species in Sabah and Sarawak from 2013 to 2017 (top). Number of malaria cases according to year and *Plasmodium* species in Peninsular Malaysia from 2013 to 2017 (bottom)

Figure 1 illustrates the number of malaria cases according to year and causative agents (*Plasmodium* species) in Sabah and Sarawak and Peninsular Malaysia from 2013 to 2017. The commonest type of *Plasmodium* species reported in Sabah and Sarawak was *P. knowlesi* with a sharp increase for 2017. There were 1468 cases in 2013 and it increased to 2252 cases in 2014. Then it reduced to 1527 cases in 2015 and 1464 cases in 2016 and eventually rose to 3191 cases in 2017. The other four types of *Plasmodium* reported fewer than 500 cases per year and showed decreasing trend year by year.

#### Peninsular Malaysia

However, there were more *P. vivax* cases (1548; 42.2%) as compared to 1478 (40.3%) for *P. knowlesi* cases notified in Peninsular Malaysia. The third common infection was by *P. falciparum* (603; 16.4%).

Figure 1 shows in contrast to Sabah and Sarawak, the commonest type of *Plasmodium* species reported from 2013 to 2016 in Peninsular Malaysia was *P. vivax* followed by *P. knowlesi* and *P. falciparum.* All three species shared the same trend. The number of malaria cases due to *P. vivax, P. knowlesi* and *P. falciparum* decreased from 2013 to 2015 and increased in 2016. There were 487 cases of *P. vivax* notified in 2013, 400 cases in 2014, 203 cases in 2015 and 284 cases in 2016. For *P. knowlesi* and *P. falciparum*, the reported cases were 474 and 268 in 2013, 332 and 103 in 2014, 113 and 70 in 2015, and 136 and 96 in 2016, respectively. However, in 2017, there was a surge in the number of notified *P. knowlesi* cases (423) in Peninsular Malaysia despite a decreasing trend for the other *Plasmodiu*m species (174 for *P. vivax* and 66 for *P. falciparum*).

### Malaria case classification and number of malaria death

#### Overall

Throughout the 5 years, indigenous cases contributed the most number of cases (13, 487; 81.7%), followed by import A cases (2916; 17.7%). Majority of the cases (16,454; 99.7%) were alive post infection (Table 3). There were 46 malaria deaths during the 5-year period. Total number of deaths in Malaysia decreased from 14 in 2013, 9 in 2014, 8 in 2015 to 2 in 2016. In spite of that, it escalated to 13 deaths in 2017 (see Table 4).

#### Sabah and Sarawak

There are differences in the number of reported malaria cases between Peninsular Malaysia and Sabah and Sarawak. In Sabah and Sarawak, more than 90% of the reported malaria were indigenous cases (11,582; 90.2%) followed by only 1218 (9.5%) import A cases. Similar percentages were observed in both regions with 12,813 (99.8%) alive patients in Sabah and Sarawak (Table 3).

Results in Table 4 reveal that Sarawak reported cases of malaria death every year from 2013 to 2017. The highest number of deaths was 3 (2013 and 2017), while the lowest was 1 (2014 and 2016). In Sabah, the number of deaths were reported every year except for year 2016. There were 3 deaths in 2013, 4 in 2014, 1 in 2015, and 4 in 2017.

#### Peninsular Malaysia

In Peninsular Malaysia the difference in the percentage was very small, with 1905 (52.0%) indigenous cases and 1698 (46.3%) import A cases. There were 3641 (99.4%) alive patients in Peninsular Malaysia. However, the percentage of death is higher (24, 0.6%) than the overall death in Malaysia which was 46 (0.3%) (Table 3).

In Peninsular Malaysia, there was no specific trend of number of malaria deaths based on year or states. Table 4 shows that in 2013, Kelantan reported 3 deaths but there was no death from 2014 to 2017. Selangor reported 2 deaths in 2014 and 2017, while there was 1 death in 2013. In 2015, Perak reported 2 deaths. In 2016, Negeri Sembilan was the only state in Peninsular Malaysia which reported malaria death (1 case). There were 2 deaths for each state of Selangor and Perak in 2017. There was no reported malaria death in Penang and Melaka over the 5-year period.

### Incidence rate, mortality rate and case fatality rate of malaria

#### Overall

**Fig. 2:**
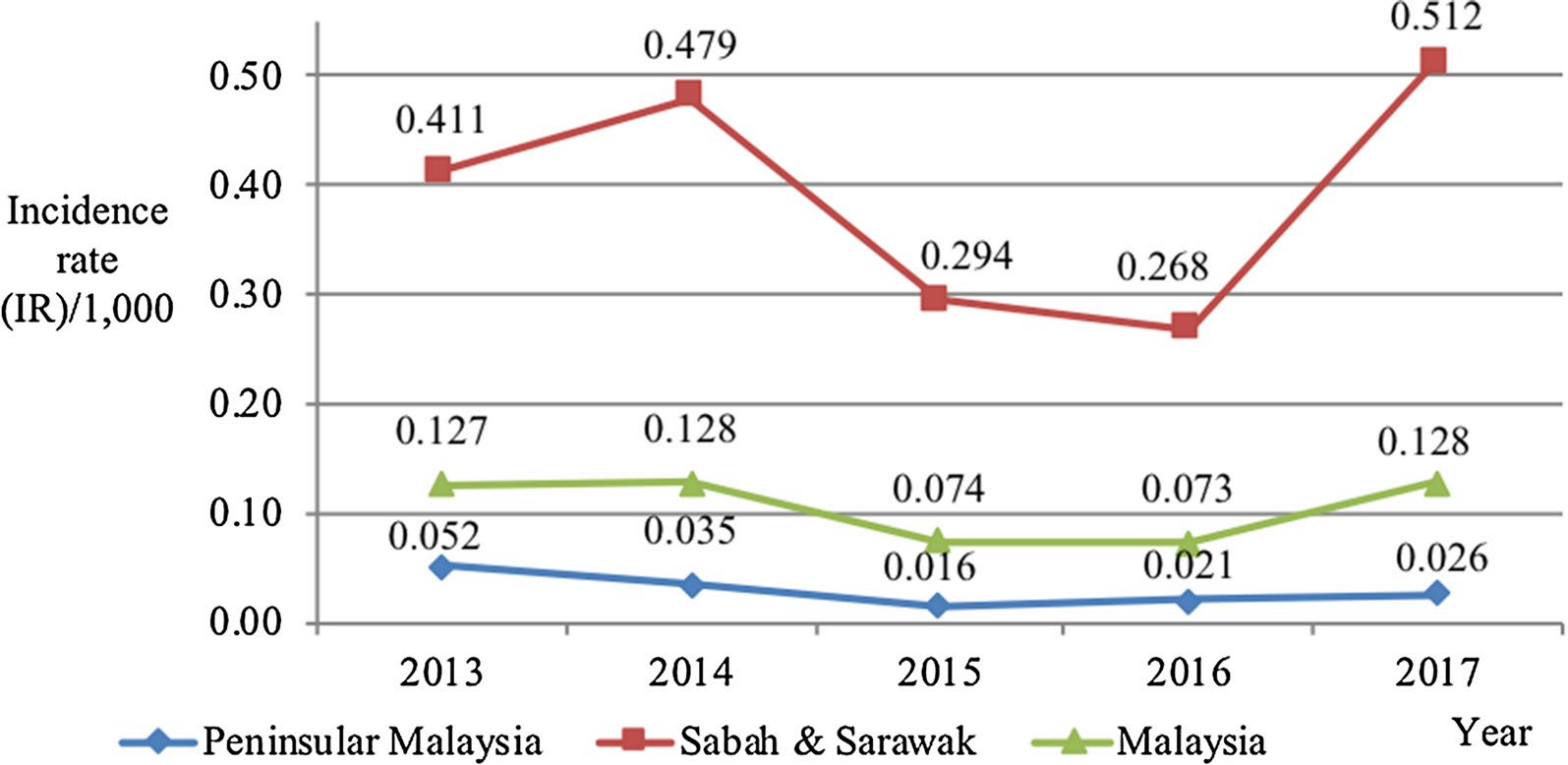
Incidence rate (IR)/1000 of malaria cases in Peninsular Malaysia, Sabah and Sarawak and whole Malaysia from 2013 to 2017

The overall average incidence rate, mortality rate and case fatality rates for malaria from 2013 to 2017 in Malaysia were 0.106 per 1000, 0.030 per 100,000 and 0.27%, respectively (Table 5). The trend in the whole country followed similar trend with Sabah and Sarawak even though the incidence rate between the years did not change very much compared to Sabah and Sarawak. Figure 2 shows that the incidence rate of malaria slightly rose from 0.127 per 1000 in 2013 to 0.128 per 1000 in 2014 and then reduced to 0.074 per 1000 in 2015 and 0.073 per 1000 in 2016 before it increased again to 0.128 per 1000 in 2017.

**Table 5:** Average incidence rate (IR)/1000, average mortality rate (MR)/100,000 and average case fatality rate (CFR) (%) of malaria according to state in Malaysia from 2013 to 2017

State	Average incidence rate/1000	Average mortality rate/100,000	Average case fatality rate (%)
Sarawak	0.420	0.074	0.18
Sabah	0.383	0.064	0.14
Kelantan	0.097	0.036	0.16
Pahang	0.064	0.050	0.76
Perak	0.043	0.032	1.48
Selangor	0.030	0.016	0.57
Negeri Sembilan	0.028	0.055	2.81
Terengganu	0.017	0.017	1.82
Johor	0.015	0.006	0.33
Penang	0.012	–	–
FT of Kuala Lumpur	0.011	0.011	1.25
Kedah	0.010	0.009	1.25
Perlis	0.007	0.083	20.00
Melaka	0.006	–	–
FT of Labuan	0.002	–	–
Malaysia	0.106	0.030	0.27

*FT* federal territory

**Fig. 3:**
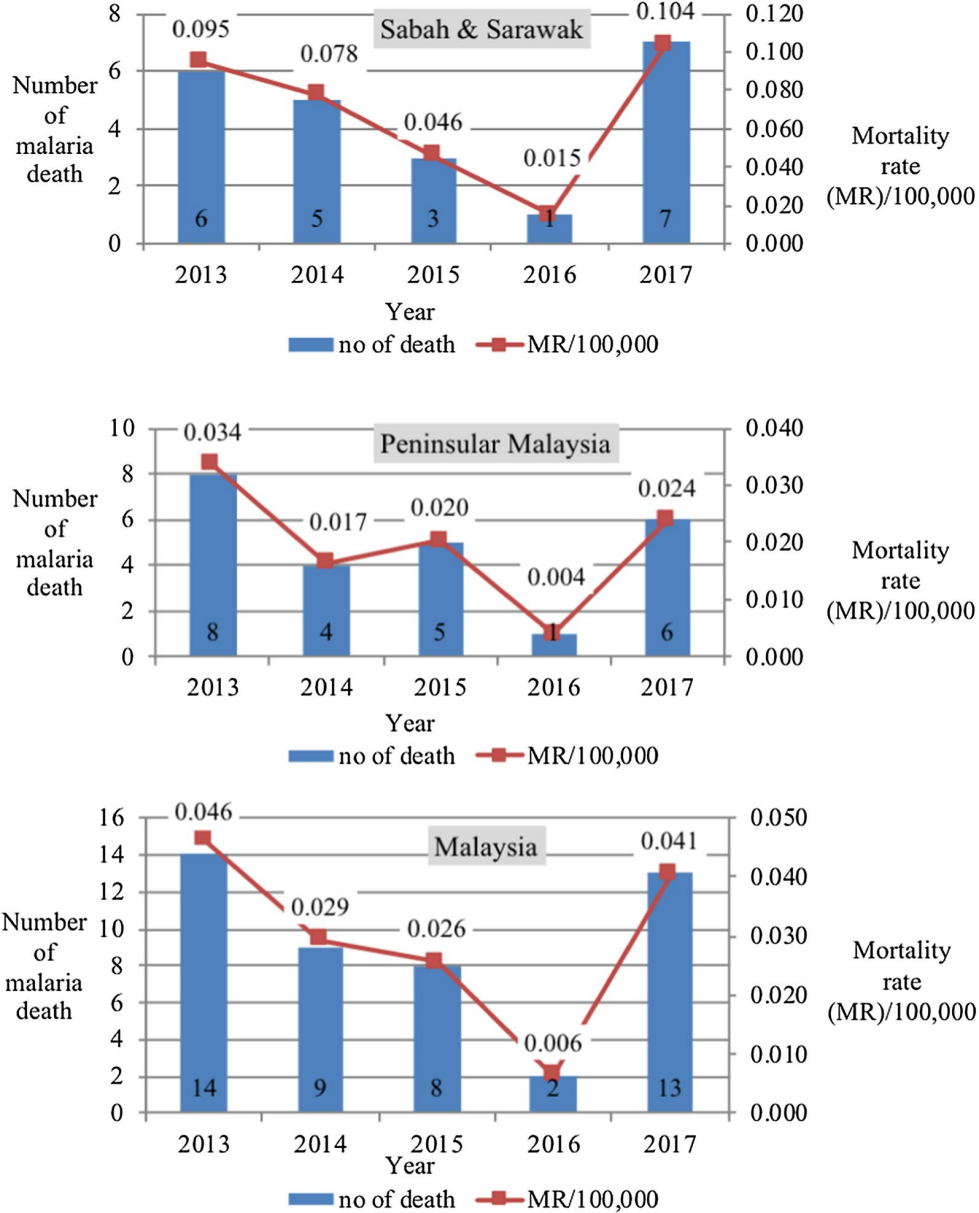
Number of malaria deaths and mortality rate (MR) in Sabah and Sarawak from 2013 to 2017 (top). Number of malaria deaths and mortality rate (MR) in Peninsular Malaysia from 2013 to 2017 (centre). Number of malaria deaths and mortality rate (MR) in Malaysia from 2013 to 2017 (bottom)

In Fig. 3, it showed that the mortality rate for malaria in Malaysia decreased from 0.046 per 100,000 in 2013 to 0.029 per 100,000 in 2014, 0.026 per 100,000 in 2015 and sharply declined to 0.006 per 100,000 in 2016. Then, it increased in 2017 to 0.041 per 100,000 populations.

#### Sabah and Sarawak

Table 5 reveals that Sarawak reported the highest average incidence rate of 0.420 per 1000 population followed closely by Sabah with an average incidence rate of 0.383 per 1000 population. Sarawak also had higher number of average mortality rate of 0.074 per 100,000 than Sabah with average mortality rate of 0.064 per 100,000 populations. Even though the average mortality rate in Sabah and Sarawak were higher than Peninsular Malaysia (except for Perlis), their average case fatality rate were lower (Sarawak; 0.18% and Sabah; 0.14%) than Malaysia (0.27%).

Generally, over the 5-year period, the incidence rate of malaria in Sabah and Sarawak consistently outnumbered the incidence rate of malaria notified in Peninsular Malaysia and also Malaysia. There were two different trends in notified number of malaria cases and incidence rate of malaria between Peninsular Malaysia and Sabah and Sarawak from 2013 to 2017 (Fig. 2). In Sabah and Sarawak, from 2013 to 2014 the incidence rate rose from 0.411 to 0.479 per 1000 populations and declined in 2015 to 2016 to 0.294 and 0.268 per 1000 populations, respectively. Then it rose up sharply to 0.512 per 1000 populations in 2017.

Similar trend for mortality rate across the 5 years was observed in Sabah and Sarawak. Figure 3 shows that the mortality rate for malaria in Sabah and Sarawak decreased steadily from 0.095 per 100,000 in 2013 to 0.078 per 100,000 in 2014, 0.046 per 100,000 in 2015 and 0.015 per 100,000 in 2016. However, it drastically shot up again in 2017 to 0.104 per 100,000 populations. Over the years, the mortality rate of malaria in Sabah and Sarawak consistently higher than the reported mortality rate in Peninsular Malaysia and Malaysia as a whole.

In Sabah and Sarawak, the average incidence rate for *P. knowlesi* cases was tenfold (0.302 per 1000) compared to *P. vivax* cases (0.036 per 1000) and *P. falciparum* cases (0.031 per 1000) (Table 6). The other *Plasmodium* species average incidence rate were 0.017 per 1000 for *P. malariae,* 0.004 per 1000 for mixed *Plasmodium* and 0.002 per 1000 for *P. ovale.*

**Table 6:** Average incidence rate (IR)/1000, mortality rate (MR)/100,000 and case fatality rate (CFR, %) of malaria according to *Plasmodium* species in Peninsular Malaysia and Sabah and Sarawak from 2013 to 2017

	Average incidence rate/1000		Average mortality rate/100,000		Average case fatality rate (%)	
	Peninsular Malaysia	Sabah and Sarawak	Peninsular Malaysia	Sabah and Sarawak	Peninsular Malaysia	Sabah and Sarawak
*P. knowlesi*	0.012	0.302	0.012	0.049	1.20	0.15
*P. vivax*	0.013	0.036	0.001	0.003	0.04	0.04
*P. falciparum*	0.005	0.031	0.006	0.016	1.41	0.39
*P. malariae*	0.0001	0.017	–	–	–	–
*P. ovale*	0.0001	0.002	–	–	–	–
Mixed *Plasmodium* infections	0.0002	0.004	0.001	–	2.50	–
Total	0.030	0.393	0.020	0.068	0.70	0.16

For average mortality rate, the similar trend was observed for Peninsular Malaysia and Sabah and Sarawak. Both recorded the highest average mortality rate in *P. knowlesi.* Sabah and Sarawak recorded 0.049 per 100,000 populations. It was followed by the average mortality rate in *P. falciparum* (0.016 per 100,000) and *P. vivax* (0.003 per 100,000). In total, Sabah and Sarawak notified higher average incidence rate (0.393 per 1000) and average mortality rate (0.068 per 100,000) than Peninsular Malaysia.

Nevertheless, the highest average case fatality rate in Sabah and Sarawak was due to *P. falciparum* infection which was 0.39% as compared to 0.15% in *P. knowlesi* and 0.04% in *P. vivax* infection. There was no death reported due to infection from *P. malariae, P. ovale* and mixed *Plasmodium* infection in Sabah and Sarawak.

#### Peninsular Malaysia

All states and federal territory in Peninsular Malaysia reported below the national average incidence rate with less than 0.100 per 1000 populations as in Table 5. The highest average incidence rate was reported in Kelantan (0.097 per 1000) followed by Pahang (0.064 per 1000), Perak (0.043 per 1000), Selangor (0.030 per 1000), Negeri Sembilan (0.028 per 1000), Terengganu (0.017 per 1000), Johor (0.015 per 1000), Penang (0.012 per 1000), FT of Kuala Lumpur (0.011 per 1000), Kedah (0.010 per 1000), Perlis (0.007 per 1000) and Melaka (0.006 per 1000).

Surprisingly, Perlis reported the highest average mortality rate and highest average case fatality rate of 0.083 per 100,000 and 20.00%, respectively for the whole Malaysia exceeding Sabah and Sarawak. It was followed by Negeri Sembilan (0.055 per 100,000) and Pahang (0.050 per 100,000). Most of the states in Peninsular Malaysia documented higher average case fatality rate than Malaysia; such as Perlis (20.0%), Negeri Sembilan (2.81%), Terengganu (1.82%), Perak (1.48%), Kedah and Federal Territory of Kuala Lumpur (1.25%), Pahang (0.76%), Selangor (0.57%) and Johor (0.33%). The only state which had lower rate than the national rate was Kelantan (0.16%).

On the other hand, the incidence rate of malaria in Peninsular Malaysia showed reduction from 0.052 per 1000 populations in 2013 to 0.035 per 1000 populations in 2014 and 0.016 per 1000 populations in 2015. Then it slightly rose to 0.021 per 1000 populations in 2016 and 0.026 per 1000 populations in 2017 (Fig. 2).

Nevertheless, the trend for mortality rate by year was not similar in Peninsular Malaysia (Fig. 3). It was 0.034 per 100,000 in 2013 and then it declined to 0.017 per 100,000 in 2014. It slightly rose to 0.020 per 100,000 in 2015 before it sharply decreased to only 0.004 per 100,000 in 2016. Unfortunately, it shot up again to 0.024 per 100,000 in 2017.

Based on the *Plasmodium* species (Table 6), the highest average incidence rate in Peninsular Malaysia was seen in *P. vivax* cases (0.013 per 1000), followed closely by *P. knowlesi* cases (0.012 per 1000) and *P. falciparum* (0.005 per 1000). For average mortality rate, a similar trend was observed for Peninsular Malaysia and Sabah and Sarawak. The highest average mortality rate was recorded in *P. knowlesi* with 0.012 per 100,000. It was followed by the average mortality rate in *P. falciparum* (0.006 per 100,000) and *P. vivax* (0.001 per 100,000). There was also death due to mixed *Plasmodium* infection in Peninsular Malaysia with an average mortality rate of 0.001 per 100,000. Peninsular Malaysia reported lower rate than Sabah and Sarawak with 0.030 per 1000 for average incidence rate and 0.020 per 100,000 for average mortality rate.

In Peninsular Malaysia, mixed *Plasmodium* infection recorded the highest average case fatality rate of 2.50%. Then it was followed by average case fatality rate in *P. falciparum* infection which was 1.41%, 1.20% in *P. knowlesi* and 0.04% in *P. vivax* infection. Unlike the total average incidence rate and total average mortality rate, the total average case fatality rate was demonstrated higher in Peninsular Malaysia (0.70%) than Sabah and Sarawak (0.16%).

## Discussion

Malaria was first recorded in Malaysia as early as the 19^th^ Century in Perak, Selangor, Pahang and Negeri Sembilan in Peninsular Malaysia [11]. The enforcement of Malaria Eradication Programme and Malaria Control Programme by the Ministry of Health, Malaysia has managed to reduce the number of notified malaria cases over recent decades from 243,870 cases in 1961 to 4725 in 2012 [2]. From the present analysis of data between 2013 and 2017, it was found that the numbers have been further reduced to 2302 in 2016 before it rose to 4114 in 2017.

The analysis also showed that Sabah state has the highest number of cases followed by the state of Sarawak for the year 2013 to 2015 and 2017. However, in 2016, Sarawak reported the highest number of malaria cases. The possible explanation for lower cases in Sabah in 2016, might be due to the strong El Nino weather phenomenon that hit Sabah in 2016 that led to changing rainfall and weather patterns which impact on the vector bionomics [8]. In general, Sabah and Sarawak contributed more than 75% of the notified malaria cases in Malaysia, whilst the top four states in Peninsular Malaysia namely Selangor, Kelantan, Perak and Pahang reported around 17% of the total malaria cases in combination. Given the high numbers of malaria cases in Sabah and Sarawak, many research and publications [5–8, 12–15] focused on the epidemiology, patterns and characteristics of notified malaria cases in these two states. Based on recent literature search, as far as is known, this is the most current analysis that described the sociodemographic profile and epidemiology of malaria cases between Peninsular Malaysia and Sabah and Sarawak.

In both regions, the majority of malaria cases occurred among males. It was suggested that male adults are at a higher risk than females of being infected with malaria because of their occupational exposure, which involves forest or plantation work, farming or agricultural work, such as in palm oil plantation work which exposes them to malaria vectors [12–14].

It was interesting to note that the cases in Sabah and Sarawak were older than those from Peninsular Malaysia. Age was indirectly related to the types of *Plasmodium* species reported. Previous studies done in Sabah had reported that age of patients with PCR-confirmed *P. knowlesi* was significantly older than that of patients infected with PCR-confirmed *P. falciparum* or *P. vivax* [5, 6, 8, 12–16]. In the current data, the majority of malaria cases in Sabah and Sarawak were *P. knowlesi* while there were more *P. falciparum* and *P. vivax* in Peninsular Malaysia, hence supporting and confirming preceding findings. Even though the reasons for the difference in ages between *Plasmodium* species was still unclear, it was suggested to be associated with occupational exposure among male, older age groups [15].

Higher numbers of notified malaria cases in Sabah and Sarawak and Peninsular Malaysia were observed in high forested states compared to the low forested states. States with high numbers of malaria cases, such as Sabah and Sarawak, known for hilly and mountainous areas with the famous Mount Kinabalu in Sabah located at the Crocker range, while Kelantan, Perak and Pahang have the highest forest density in Peninsular Malaysia. It was found that there is a significant positive correlation between forest density and number of malaria cases [11]. The amount of forest is important as it increases contact between human hosts and *Anopheles* mosquitoes’ habitats and increases chances for malaria transmission. There was also an increase in malaria cases due to the opening of new rubber estates through the clearing of previously thick forests, the building of new roads and settlements for the labourers [11]. Furthermore, in Peninsular Malaysia, the indigenous people still live in many forested areas in Kelantan, Perak and Pahang [17]. Studies done in Peninsular Malaysia have shown that malaria is common among the indigenous people [18, 19]. This study showed that there are more indigenous people (i.e., Orang Asli) infected with malaria in Peninsular Malaysia than in Sabah and Sarawak which mostly involved their local Pribumis.

On the other hand, in urbanized state in Peninsular Malaysia such as Selangor, malaria cases were seen more in foreign workers from endemic countries such as Myanmar, Bangladesh and Indonesia. In Malaysia, foreign workers work in six main sectors: manufacturing, construction, agriculture, plantations, mining, quarrying, and the service sector [20]. Most are at higher risk of contracting malaria vector due to occupational exposure which involves mainly forestry and agriculture. Data showed a higher percentage of foreigners and import A cases in Peninsular Malaysia than in Sabah and Sarawak.

According to *Plasmodium* species, reduction in the incidences of *P. falciparum* and *P. vivax* were seen in Peninsular Malaysia and Sabah and Sarawak. However, there has been an apparent recent increase in the incidence of malaria from the simian parasite *P. knowlesi,* especially in Sabah and Sarawak.

The first naturally acquired case of *P. knowlesi* in humans in Malaysia was reported in Pahang in 1965 followed by a second probable case in Johor a few years later [4]. Both states are in Peninsular Malaysia. Compared to other malaria species, knowlesi malaria was thought to be a rare disease until a large focus of human infection was described in Kapit, Sarawak in 2004 [21]. Then the number of cases in Sabah appeared to increase, mainly in the interior region adjacent to Sarawak, before it spread to the other parts of Sabah [5]. Since then, the number of knowlesi cases has increased in Sabah and Sarawak but not in Peninsular Malaysia [5, 6, 8, 12–14, 22, 23].

Several possible reasons for the emergence of *P. knowlesi* especially in Sabah have been postulated. Firstly, due to the high cases of knowlesi cases in Sabah, the microscopy skill of laboratory staff to recognize the species has increased and this may account for the increase in reporting of the species [5]. Microscopically, *P. knowlesi* infection may mimic *P. malariae* infection and other malaria infections (*P. falciparum* and *P. vivax*) [24]. In order to confirm that the infection is due to knowlesi malaria, polymerase chain reaction (PCR) test plays a major role. Since improving microscopy training will not help to distinguish *P. knowlesi* from *P. malariae* which are indistinguishable on routine microscopy, in Sabah, PCR test is routinely done for all *P. knowlesi* and *P. malariae* cases since 2010. Molecular detection was further extended to Sarawak and Peninsular Malaysia in 2011. For other species (*P. falciparum, P. vivax* and *P. ovale)*, 10% from the positive microscopy slides will be sent to either Public Health or State Vector Laboratories for re-examination. If there is any discordant result, PCR test will be done to confirm the *Plasmodium* species.

In addition, people are at risk of re-infection of *P. knowlesi* due to lack of immunity to the species [15, 25]. Environmental change due to extensive deforestation in Sabah has disrupted mosquito vectors’ and simian hosts’ habitat as well as more interaction with the humans [26]. This is supported by the geographic distribution of knowlesi cases in Sabah which are concentrated in the forested areas [5, 6]. It has also been suggested that the increase in *P. knowlesi* infection in Malaysia could be due to waning immunity to human malaria, subsequent to the control of *P. falciparum* and *P. vivax* [27].

In general, the mortality rate in Malaysia is lower than the rate reported by WHO in 2017 which were 11.7 per 100,000 globally and 1.2 per 100,000 in Southeast Asia [1]. Over the 5-year period (2013 to 2017), the mortality rates in Sabah and Sarawak have exceeded the national mortality rate while the mortality rate in Peninsular Malaysia is lower than Malaysia’s mortality rate. Furthermore, the average mortality rate by species in Peninsular Malaysia and Sabah and Sarawak reported the highest rate in knowlesi cases followed by falciparum and vivax malaria cases. Published studies have revealed that *P. knowlesi* which is prevalent in Sabah and Sarawak has a higher risk of causing severe malaria compared to the other *Plasmodium* species even at a low parasitaemia level [4, 8]. It was reported that *P. knowlesi* had a significant higher risk of severity than *P. falciparum* with a 2.96-fold with a *p* value of 0.020 [15]. They found that severe malaria occurred in 29% of patients infected with *P. knowlesi*, 16% in *P. vivax* and 11% in *P. falciparum* infection. The common complications seen are respiratory distress, acute renal failure and shock [28]. *Plasmodium knowlesi* is noted to be an important cause of severe and fatal malaria in Sabah [4, 15, 28–30].

In contrast, *P. falciparum* has the highest average case fatality rate, followed by *P. knowlesi* and *P. vivax.* A study done in Sabah published similar report in which during 2010–2014, their case fatality rate was also the highest in *P. falciparum* cases with 4.83 deaths/1000 cases followed by 3.08 deaths/1000 in *P. knowlesi* cases and the lowest was reported in *P. vivax* cases with 0.87 deaths/1000 cases [23].

Nevertheless, based on mortality by state, Perlis is one of the states in Peninsular Malaysia reported with the highest average mortality rate and case fatality rate in Malaysia. Perlis notified the least number of cases in Malaysia with only 9 cases and 1 death during the 5-year period. It involved a 26 years old male foreigner who was infected with *P. falciparum* (import A case). Further history and clinical details on this patient was not available in the dataset. Possible explanation should be death due to severe falciparum malaria.

Unlike incidence and mortality rates, Sabah and Sarawak have lower average case fatality rates compared to Malaysia and most of the states in Peninsular Malaysia. Previous published report also showed declining case fatality rate in Sabah [4, 30]. It stated that the number of deaths due to *P. knowlesi* in Sabah has remained relatively stable despite the increase in the number of notified cases. This indicates the improvement in the ability to detect severe knowlesi malaria at the early stage and the institution of proper management of severe knowlesi malaria with the increase use of intravenous artesunate [15, 23] following the extensive research done on *P. knowlesi* in Sabah. The Management Guidelines of Malaria in Malaysia published in 2013 recommend intravenous artesunate for all patients with severe malaria caused by any *Plasmodium* species [31]. It was adopted by WHO and the recent WHO guidelines recommend intravenous artesunate to be used for all patients with knowlesi malaria and more than 100,000 parasites/μL of blood or, if testing for laboratory criteria for severe malaria is not available, more than 20,000 parasites/μL blood is indicated [32–34]. However, even though the case-fatality rate is relatively low, the ongoing increase in notification of *P. knowlesi* cases in Sabah and Sarawak highlights the possibility of an increase in the number of deaths in the coming years [15].

In terms of control, vector management approaches, such as insecticide spray and use of insecticide-treated bed nets, have contributed greatly to the successful control and reduction in number of human malaria cases in Malaysia. Unfortunately, looking at the current trend of malaria cases in Malaysia, it shows that the interventions need to be redesigned in order to effectively control zoonotic *P. knowlesi* cases. In view of the different dominant species of *Plasmodium,* customized prevention and control programmes should be focused for Peninsular Malaysia and Sabah and Sarawak. Malaysia is on track to meet the elimination targets for the human-only species malaria but while-ever the monkey reservoir, mosquitoes and humans coexist, *P. knowlesi* malaria would not be eliminated in this timeline.

The current vector control strategy should be enhanced and maintained in Peninsular Malaysia. In view of the increasing trend of knowlesi malaria in Peninsular Malaysia and Sabah and Sarawak, additional measures are required in both regions. Better surveillance is needed. Improved diagnostic capacity in detecting *P. knowlesi* species microscopically and the use of molecular diagnosis has been made available nationally starting from the year 2010 in Sabah and 2011 for Sarawak and Peninsular Malaysia. It is one of the strengths of the Malaria Control Programme and probably should be extended to other species (*P. falciparum, P. vivax* and *P. ovale*) including to the other surrounding knowlesi endemic countries as well. In addition, complete geographic distribution maps involving the *Plasmodium* species especially *P. knowlesi* is very important. Understanding the geographical distribution of *P. knowlesi* is another key point for identifying high-risk infection areas and designing proper control strategies and surveillance systems. It is also vital for projecting areas where malaria transmission is likely to occur even after human malarias have been controlled.

In Sabah, *Anopheles balabacensis* is the primary vector of *P. knowlesi*. It is found typically in village, forest and farming sites in which it interacts with humans easily through daily human activity [35]. It rests and feeds outdoors (exophagic) especially after dusk. It had been shown that indoor residual spraying (IRS) of houses has an independent protective effect against knowlesi infection in human [14]. However, due to the outdoor biting and resting behaviour of the mosquitoes, it may hinder the effectiveness of IRS and treated bed nets [35, 36]. Currently, there is an ongoing study in Sabah and Sarawak looking into the effectiveness of outdoor residual spray (ORS), in collaboration between Institute of Medical Research and Ministry of Health, Malaysia [37]. At the individual level, intensification of health education on malaria and the emphasis on the use of personal protection which include wearing proper clothing with long sleeve, insect repellent and mosquito coil when entering the high-risk area for the people at risk may play a role in reducing the transmission.

In the present analysis, secondary data on malaria notification in Malaysia was used as a primary data source. The use of malaria notification has its own limitation in estimating the incidence since it may lead to underestimation of true malaria incidence, given that a possibility of malaria cases goes un-notified. However, through a mandatory notification of malaria and the increase recognition of malaria, especially knowlesi malaria over recent years may have changed reporting practices. Secondly, the use of secondary data also restricts the detail information needed for further in-depth analysis of individual cases. There was no collection of information on specific risk factors such as an occupational exposure or where patients acquired their malaria infection.

Microscopically, the appearance of *Plasmodium* parasite may mimic between each other species. The absence of detailed information with regards to percentage of malaria cases confirmed by microscopy, a rapid test or a molecular diagnostic test imposed a quality limitation to clinical data collection and have led to limited analyses in this study. Nevertheless, all the cases included in this analysis have been rectified and only malaria confirmed cases have been analysed. In reporting the demographic and geographic features of malaria, some analyses were reported as aggregation of all *Plasmodium* species despite the marked decline in *P. falciparum* and *P. vivax* and marked rise in *P. knowlesi* over this time period. Important between-species differences may be lost amongst this aggregation.

## Conclusions

The present analysis recorded different trends and characteristics of notified malaria cases in Peninsular Malaysia and Sabah and Sarawak. Current vector control and interventions are successful in the reduction of the human only *Plasmodium* infection. Nevertheless, more intervention and research are still needed to focus on the control and prevention of the emerging zoonotic *P. knowlesi* infection. These differences highlight the need to customised prevention and control measures in Peninsular Malaysia and East Malaysia (Sabah and Sarawak).

## Data Availability

The datasets used and/or analysed during the current study are available from the corresponding author on reasonable request.

## Abbreviations

CFR: Case fatality rate
IR: Incidence rate
IRS: Indoor residual spray
MR: Mortality rate
MREC: Medical research ethics committee
NMRR: National medical research register
ORS: Outdoor residual spray
PCR: Polymerase chain reaction
WHO: World Health Organization
